# Interaction between *O*-GlcNAc Modification and Tyrosine Phosphorylation of Prohibitin: Implication for a Novel Binary Switch

**DOI:** 10.1371/journal.pone.0004586

**Published:** 2009-02-24

**Authors:** Sudharsana R. Ande, Saby Moulik, Suresh Mishra

**Affiliations:** 1 Department of Internal Medicine, University of Manitoba, Winnipeg, Manitoba, Canada; 2 Department of Physiology, University of Manitoba, Winnipeg, Manitoba, Canada; University of Queensland, Australia

## Abstract

Prohibitin (PHB or PHB1) is an evolutionarily conserved, multifunctional protein which is present in various cellular compartments including the plasma membrane. However, mechanisms involved in various functions of PHB are not fully explored yet. Here we report for the first time that PHB interacts with *O*-linked β-*N*-acetylglucosamine transferase (*O*-GlcNAc transferase, OGT) and is *O*-GlcNAc modified; and also undergoes tyrosine phosphorylation in response to insulin. Tyrosine 114 (Tyr114) and tyrosine 259 (Tyr259) in PHB are in the close proximity of potential *O*-GlcNAc sites serine 121 (Ser121) and threonine 258 (Thr258) respectively. Substitution of Tyr114 and Tyr259 residues in PHB with phenylalanine by site-directed mutagenesis results in reduced tyrosine phosphorylation as well as reduced *O*-GlcNAc modification of PHB. Surprisingly, this also resulted in enhanced tyrosine phosphorylation and activity of OGT. This is attributed to the presence of similar tyrosine motifs in PHB and OGT. Substitution of Ser121 and Thr258 with alanine and isoleucine respectively resulted in attenuation of *O*-GlcNAc modification and increased tyrosine phosphorylation of PHB suggesting an association between these two dynamic modifications. Sequence analysis of *O*-GlcNAc modified proteins having known *O*-GlcNAc modification site(s) or known tyrosine phosphorylation site(s) revealed a strong potential association between these two posttranslational modifications in various proteins. We speculate that *O*-GlcNAc modification and tyrosine phosphorylation of PHB play an important role in tyrosine kinase signaling pathways including insulin, growth factors and immune receptors signaling. In addition, we propose that *O*-GlcNAc modification and tyrosine phosphorylation is a novel previously unidentified binary switch which may provide new mechanistic insights into cell signaling pathways and is open for direct experimental examination.

## Introduction

Prohibitin (PHB, also known as PHB1) and its homologue PHB2 are evolutionarily conserved proteins and have been shown to be involved in cell cycle progression, cell differentiation, gene transcription and mitochondrial functions [1]. However, mechanisms involved in various functions of PHBs are poorly characterized. PHB has been reported to be present in the plasma membrane especially in the lipid rafts of B cells, Caco-2 cells (a model intestinal epithelial cell), adipocytes, and endothelial cells of adipose tissue vasculature [1]–[5]. Lipid rafts has been known to serve as platforms for receptor tyrosine kinase signaling including insulin and B cell receptor signaling [6]. This involves tyrosine phosphorylation of various signaling intermediates and tyrosine phosphorylation dependent protein-protein interactions [7], [8]. PHB has been shown to be required for Ras-induced Raf-MEK-ERK activation in epidermal growth factor receptor signaling pathway however mechanism involved in this process remains to be determined [9]. Central and C-terminus part of PHB contain tyrosine residues (Tyr114 and Tyr259) which exist within a motif similar to tyrosine known to be phosphorylated in the epidermal growth factor and insulin receptors [10], in beta integrins [11] and in B cell receptors [12]. Phosphorylation of tyrosine residues within these motifs are known to be recognized by phospho tyrosine binding (PTB) domain of signaling proteins [13], [14]. Tyr114 and Tyr259 residues in PHB are highly conserved across species suggesting a functional role for these residues [1].

In addition to tyrosine phosphorylation, *O*-GlcNAc (*O*-linked β-*N*-acetylglucosamine) modification of tyrosine kinase signaling intermediates by *O*-GlacNAc transferase (OGT) has been shown to play a regulatory role in receptor tyrosine kinase signaling pathways [15]–[16]. OGT transfers *N*-acetylglucosamine from UDP-GlcNAc to a hydroxyl oxygen of serine or threonine in a β confirmation (β-*O*-glycosylation) [17]. OGT itself undergo tyrosine phosphorylation which enhances OGT activity [15]. In many proteins a yin-yang relationship (i.e. same serine/threonine residues are subjected to both phosphorylation and *O*-GlcNAc modification under different conditions) exists [18]–[20], therefore it is not surprising that they influence and regulate each other because both modifications occur at the same residue. It is not known whether tyrosine phosphorylation can also influence *O*-GlcNAc modification and *vice versa*. Here we report that PHB interact with OGT both *in vivo* and *in vitro* and is an *O*-GlcNAc modified protein. In addition, we provide data which suggests that *O*-GlcNAc modification and tyrosine phosphorylation in a protein interact with each other. Implication of this study extends beyond protein specific binary switch and offers new mechanistic insights into tyrosine kinase signaling pathways.

## Results

### PHB interacts with OGT and is an O-GlcNAc modified

**Figure 1:**
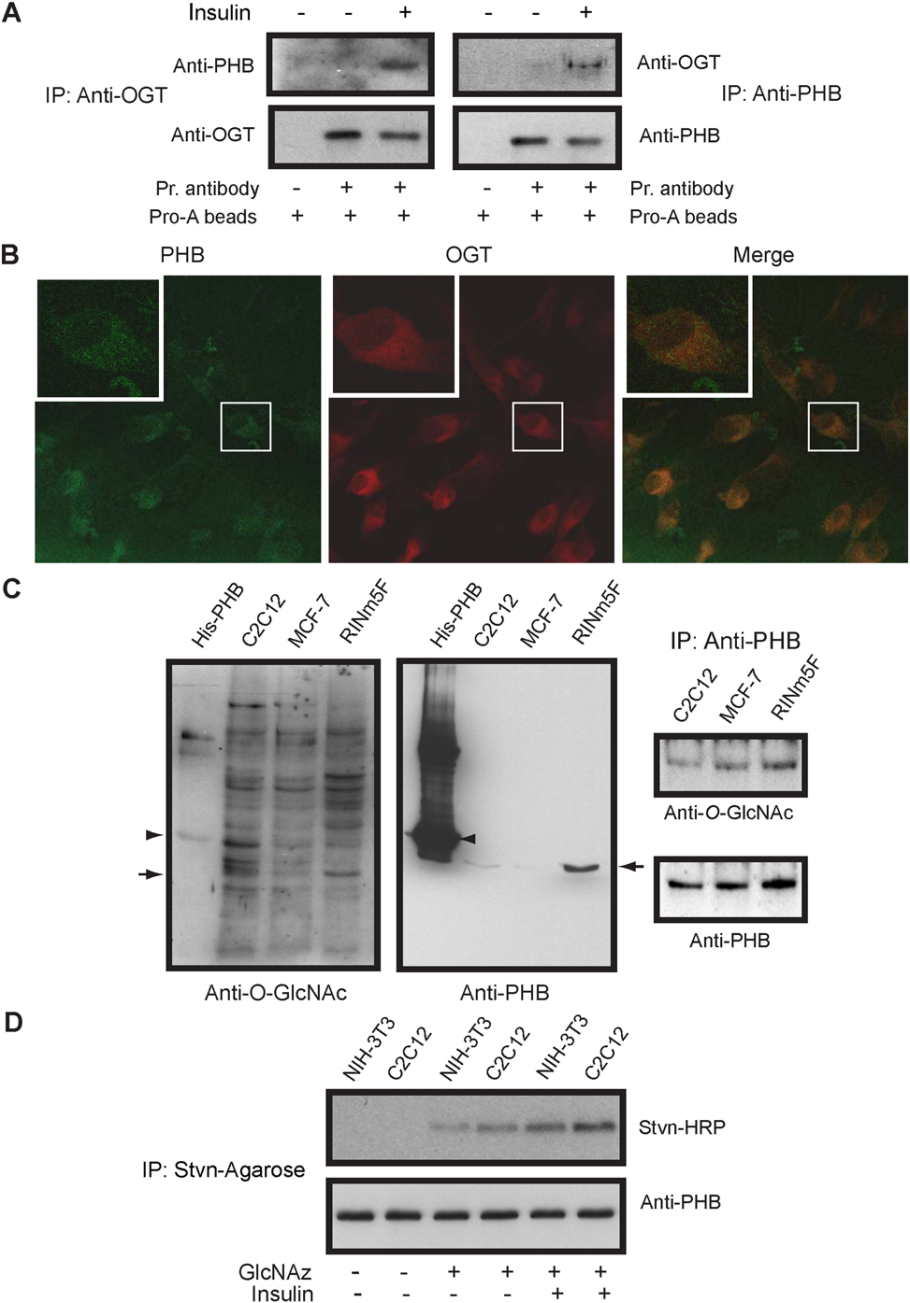
PHB interacts with OGT and is *O*-GlcNAc modified. (A) showing co-immunoprecipitation of PHB and OGT with each other. PHB and OGT co-immunoprecipitated from RINm5F cells with (100 nM) or without insulin treatment and immunoprecipitates were analyzed by immunoblotting using respective antibodies (left panel). In (B), *in situ* colocalization of PHB (green) and OGT (red) is shown in RINm5F cells by confocal microscopy. Cells were first incubated with rabbit anti-PHB (1∶100) and mouse anti-OGT (1∶500) antibodies and subsequently detected by FITC- and TRITC-conjugated secondary antibodies respectively. In (C), left panel showing anti-*O*-GlcNAc and anti-PHB immunoblots showing endogenous (__▸) and recombinant PHB (▸) as an *O*-GlcNAc modified protein. In the right panel same is shown after immunoprecipitation with anti-PHB antibody. In (D) *O*-GlcNAc metabolic labeling of PHB by GlcNAz sugar is shown. Cells were grown in the presence of azido-sugar for 72 hrs and then treated with or without insulin (100 nM). Subsequently azido sugar labeled proteins were ligated with biotin alkene and PHB was immunoprecipitated using anti-PHB antibody. Immunoprepitates were analyzed by gel-electrophoresis and immunoblotting using streptavidin-HRP conjugate and anti-PHB antibody. Representative blots of three or more experiments are shown. (Pr - primary, Pro-A - protein-A; IP - immunoprecipitation, Stvn-HRP - streptavidin horse radish peroxidase; GlcNAz - tetraacetylated azide-modified *N*-acetylglucosamine).

Because PHB and OGT both interact with insulin receptor and undergo tyrosine phosphorylation [15], [21], we examined whether PHB could interact with OGT. In co-immunoprecipitation assays, we found that both PHB and OGT co-immunoprecipitated with each other in response to insulin (Fig. 1A). A very weak signal was observed in the absence of insulin. This is further confirmed by *in situ* co-localization of PHB and OGT using confocal microscopy (Fig. 1B). This prompted us to examine whether PHB is subjected to modification by OGT. Cell lysates prepared from three different cell lines were analyzed by SDS-PAGE and processed for immunoblotting using monoclonal anti-*O*-GlcNAc antibody. Multiple *O*-GlcNAc modified protein bands were observed around expected ∼30 kDa PHB band. To confirm whether one of them is PHB, membranes were stripped and reprobed with anti-PHB antibody. Indeed one of the *O*-GlcNAc modified band exactly coincided with PHB band in overlay suggesting that PHB undergoes *O*-GlcNAc modification (Fig. 1C). Recombinant His-PHB was also detected by anti-*O*-GlcNAc antibody. A similar result was found when PHB was immunoprecipitated and analyzed by gel-electrophoresis and immunoblotting using monoclonal anti-*O*-GlcNAc antibody (Fig. 1C). *O*-GlcNAc modification of PHB was further confirmed by metabolic labeling of PHB with tetraacetylated azide-modified *N*-acetylglucosamine (GlcNAz) in C2C12 and NIH-3T3 cell lines and subsequent tagging with biotin alkyne (Fig. 1D). In both cell lines used, PHB was found labeled with GlcNAz sugar suggesting that *O*-GlcNAc modification of PHB is not cell specific (Fig. 1D). In insulin treated cells a more intense *O*-GlcNAc modified PHB band was observed in comparison to untreated controls suggesting that insulin facilitates *O*-GlcNAc modification of PHB. A similar result was found with RINm5F cells (data not shown). These data together suggest that PHB interacts with OGT and is an *O*-GlcNAc modified protein.

### O-GlcNAc modification and tyrosine phosphorylation affects each other

**Figure 2:**
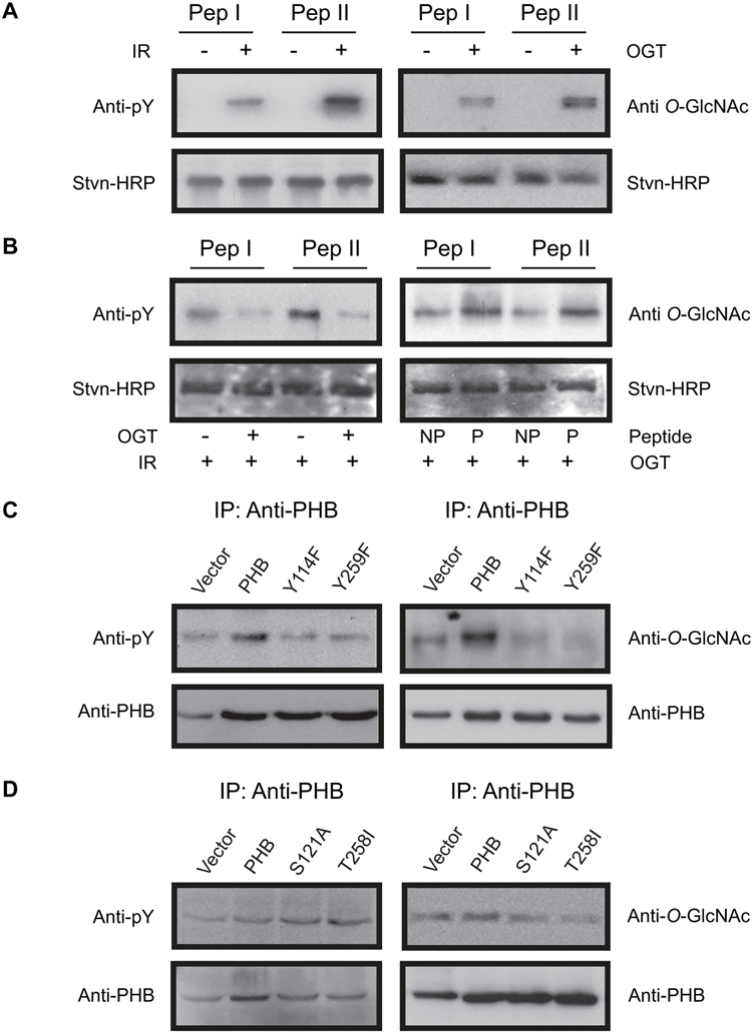
Interaction between *O*-GlcNAc modification and tyrosine phosphorylation of PHB. In (A) showing *in vitro* phosphorylation (left panel) and *O*-GlcNAc modification of PHB peptides (right panel) using insulin receptor and immunoprecipitated OGT respectively. In (B) effect of tyrosine phosphorylation on *O*-GlcNAc modification and *vice versa* are shown. To determine the effect of tyrosine phosphorylation on *O*-GlcNAc modification, PHB control peptides and phosphate blocked Tyr114 and Tyr259 containing peptides were incubated with OGT and UDP-GlcNAc and subsequently analyzed by gel-electrophoresis and immunoblotting as described in Methods (right panel). To determine the effect of *O*-GlcNAc modification on tyrosine phosphorylation, biotinylated-PHB peptides were first immobilized on streptavidin-agarose and then used for *in vitro O*-GlcNAc assays using OGT and UDP-GlcNAc. After washing (3×) in reaction buffer same peptides were used for *in vitro* kinase assays using catalytic domain of insulin receptor and ATP. Finally, peptides were analyzed by gel-electrophoresis and immunoblotting using anti-phospho tyrosine (left panel). (C and D) Effect of PHB mutants on tyrosine phosphorylation and *O*-GlcNAc modification of PHB are shown. PHB and OGT were immunoprecipitated from insulin (100 nM for 20 mts) treated RINm5F cells after 72 hrs of co-transfection with OGT and PHB/PHB mutants (Y114F, S121A, T258I and Y259F). Immunoprecipitates were analyzed with gel-electrophoresis and immunoblotting using anti-phosphotyrosine and anti-*O*-GlacNAc monoclonal antibodies. Representative blots of three or more experiments are shown. (Pep I - peptide I - pep II, peptide II, IR - insulin receptor, P - with phosphate group attached to tyrosine, NP - without phosphate group, IP - immunoprecipitation, pY - phosphotyrosine).

Because PHB undergoes both *O*-GlcNAc modification and tyrosine phosphorylation, we asked the question whether these modifications occur in neighboring residues and affect each other. Analysis of PHB protein sequence by Human Protein Reference Database, NetPhos and Scansite servers identified Tyr114 and Tyr259 as potential sites of phosphorylation. Interestingly two (i.e. Ser121 and Thr258) out of three potential *O*-GlcNAc sites (i.e. Ser101, Ser121 and Thr258) predicted (discussed later) in PHB are either adjacent to or in the close proximity of tyrosine phosphorylation sites (Tyr114 and Tyr259). Therefore, we used two synthetic peptides (Pep I - Biotin-^105^RIFTSIGED**Y**DERVLP**S**IT^123^-NH2 and Pep II - Biotin-^250^QLSRSRNI**TY**LPAGQSVLL^268^-NH2) biotinylated at the N-terminus and amidated at the C-terminus spanning tyrosine phosphorylation and *O*-GlcNAc modification sites in PHB. In *in vitro* kinase assays using catalytic domain of insulin receptor (Upstate Biotechnology, NY, USA) and ATP (Sigma, ON, Canada) and subsequent analysis with immunoblotting we found that both peptides were phosphorylated at tyrosine residue (Fig. 2A). To determine the potential *O*-GlcNAc modification of tyrosine neighboring residues, biotinylated synthetic peptides of PHB were incubated with immunoprecipitated OGT and UDP-GlcNAc and subsequently analyzed with gel-electrophoresis and immunoblotting. *O*-GlcNAc modification was detected by immunoblotting using monoclonal anti *O*-GlcNAc antibody (Fig. 2A) suggesting that they had undergone *O*-GlcNAc modification. Because both peptides contain multiple serine and/or threonine residues therefore *O*-GlcNAc modification of these peptides at sites other than Ser121 and Thr258 may not be ruled out.

To determine whether tyrosine phosphorylation modulates *O*-GlcNAc modification we used biotinylated synthetic peptides with a phosphate group attached to Tyr114 and Tyr259 in *in vitro O*-GlcNAc assays. Presence of phosphate at tyrosine residues (Tyr114 and Tyr259) significantly enhanced the *O*-GlcNAc modification of both peptides in comparison to non-phosphorylated control peptides (Fig. 2B). Similar results were found when peptides were phosphorylated using catalytic domain of insulin receptor (data not shown). This is in contrast to yin-yang sites in which phosphorylation at serine or threonine residues has been shown to be associated with down regulation of *O*-GlcNAc modification and *vice versa* [18]–[19]. To determine whether *O*-GlcNAc modification affect tyrosine phosphorylation, biotinylated peptides (Pep I and Pep II) were immobilized on streptavidin-agarose beads and processed for *in vitro O*-GlcNAc assays using immunoprecipitated OGT and UDP-GlcNAc. After washing, immobilized control peptides (without any modification) and OGT induced *O*-GlcNAc modified peptides were used for *in vitro* kinase assays using catalytic domain of insulin receptor and ATP. Interestingly tyrosine phosphorylation of these peptides was attenuated in the presence of *O*-GlcNAc modification in comparison to non-glycosylated control peptides (Fig. 2B). Taken together, these results suggest that tyrosine phosphorylation enhances *O*-GlcNAc modification whereas *O*-GlcNAc modification attenuates tyrosine phosphorylation at least in these peptides.

### Loss of tyrosine phosphorylation in PHB enhances OGT activity

**Figure 3:**
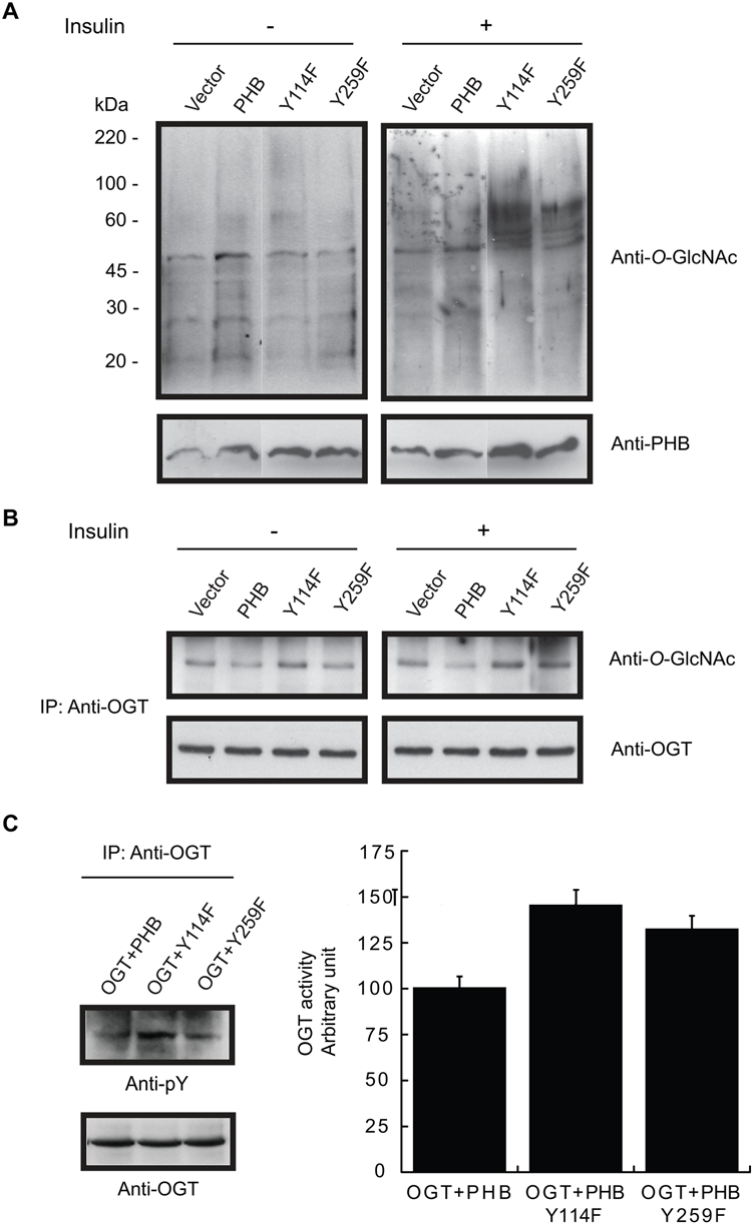
Enhanced tyrosine phosphorylation of OGT and OGT activity in cells transfected with PHB mutants (Y114F and Y259F) in response to insulin. (A) Effect of PHB/PHB mutant constructs on *O*-GlcNAc modification of various proteins in response to insulin in RINm5F cells is shown. In (B), cells were transfected with various PHB vectors and subsequently treated with or without insulin. OGT was immunoprecipitated using anti-OGT antibody and analyzed using anti-*O*-GlcNAc and anti-OGT antibodies. (C) RINm5F cells were co-transfected with OGT and PHB constructs. After 48 hrs serum starved cells were treated with insulin (100 nM) for 20 minutes. OGT was immunoprecipiataed using anti-OGT antibody and divided into two halves. One half was used for immunoblotting using anti-phospho tyrosine antibody (left panel). Another half was used for *in vitro O*-GlcNAc assay using PHB synthetic peptide II as a substrate and UDP-GlcNAc. Modified substrate was analyzed by immunoblotting using anti-OGT antibody. Subsequently immunoblots were analyzed by densitometry using NIH image analysis software. Normalized OGT activity is shown in the lower panel. Representative blots of three or more experiments are shown. Experiments were repeated for three times. (IP - immunoprecipitation, pY - phosphotyrosine).

**Figure 4:**
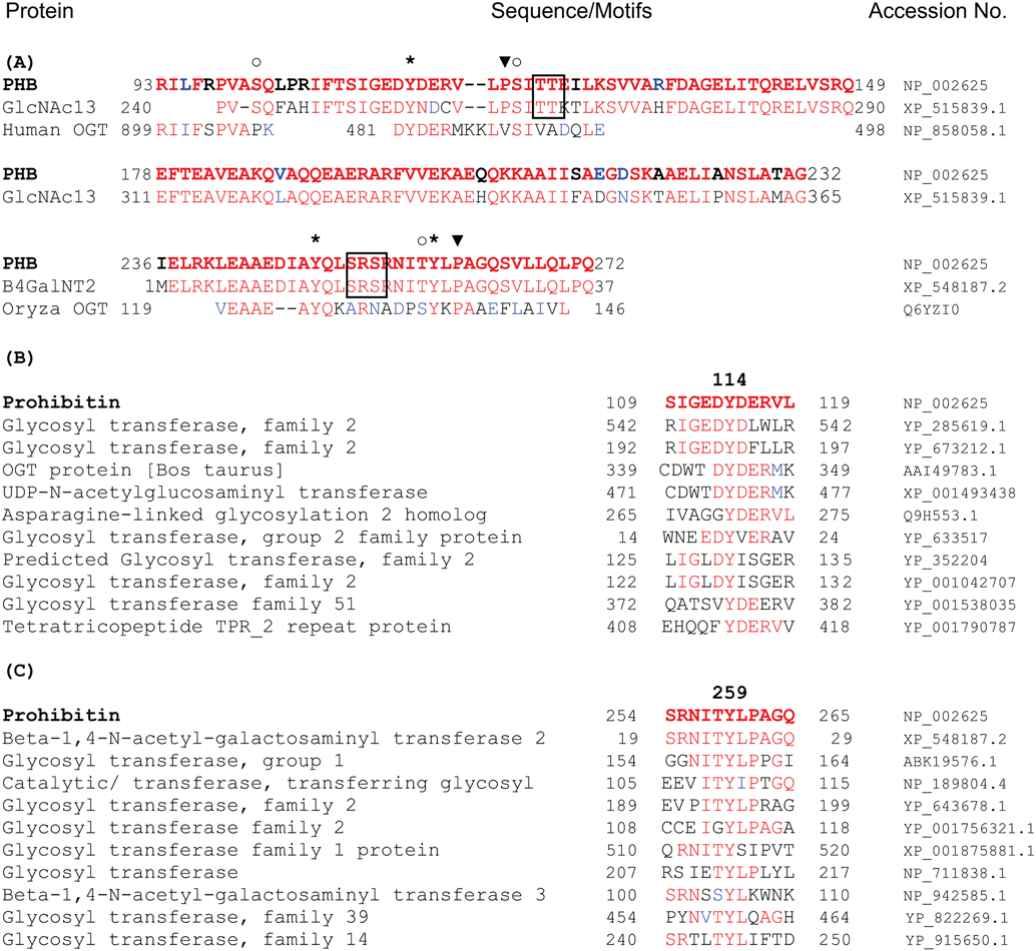
PHB and OGT family members contain similar tyrosine motifs. Multiple sequence alignments of PHB with OGT family members, as well as tyrosine motifs of PHB containing Tyr114 and Tyr259 with various OGT family members having similar motifs are shown. (A) showing sequence homology of human PHB with GlcNAc13, 4GalNT2, human OGT and Oryza OGT. Potential sites of tyrosine phosphorylation (*), *O*-GlcNAc modification (○) and proline residues (▾) at −1 and/or +3 position from potential O-GlcNAc sites are shown. Multiple serine/threonine residues in the vicinity of potential *O*-GlcNAc sites are shown in the box (□). (B) showing motif containing Tyr114, and (C) showing motif containing Tyr259 in PHB. Conserved residues are shown in red and identical residues are shown in blue. (GlcNAc13 - UDP-*N*-acetyl-alpha-D-galactosamine: polypeptide *N*-acetylgalactosaminyltransferase 13, 4GalNT2 – β-1,4-*N*-acetyl-galactosaminyl transferase 2).

To determine whether tyrosine phosphorylation and *O*-GlcNAc modification of PHB interact within cells in the same manner as in *an in vitro* assay; Tyr114, Ser121, Thr258 and Tyr259 were substituted with phenylalanine, alanine, isoleucine and phenylalanine respectively by site-directed mutagenesis. Cells were transfected with wild type and PHB mutant vectors. After insulin treatment, PHB was immunoprecipitated and subsequently analyzed with gel electrophoresis and immunoblotting. An attenuation of tyrosine phosphorylation and *O*-GlcNAc modification was observed in Tyr114Phe and Tyr259Phe PHB mutants in comparison to wild type PHB in response to insulin (Fig. 2C) suggesting that Tyr114 and Tyr259 in PHB undergo phosphorylation in response to insulin and phosphorylation at these tyrosine residues facilitates *O*-GlcNAc modification of PHB. While substitution of Ser121 and Thr258 in PHB with alanine and isoleucine respectively resulted in reduced *O*-GlcNAc modification and increased tyrosine phosphorylation of PHB in response to insulin (Fig. 2D) suggesting that *O*-GlcNAc modification attenuates tyrosine phosphorylation of PHB. No difference was observed in the absence of insulin (data not shown). Surprisingly a significant up regulation of *O*-GlcNAc modification of some proteins in the molecular weight range of ∼50–100 kDa was also observed in response to insulin in cells transfected with Tyr114Phe and Tyr259Phe PHB mutants (Fig. 3A). Next we sought to know whether OGT itself is *O*-GlcNAc modified in cells transfected with tyrosine PHB mutants. To determine this, OGT was immunoprecipitated from untreated and insulin treated cells transfected with PHB tyrosine mutants and analyzed by immunoblotting using anti-*O*-GlcNAc antibody. An enhanced *O*-GlcNAc modification of OGT was observed in cells transfected with PHB mutants in comparison to wild type PHB in response to insulin only (Fig. 3B). To gain further insight into the mechanism involved, PHB sequence was analyzed with pBLAST search for any potential homology with OGT family members. Interestingly two OGT family members (UDP-*N*-acetyl-alpha-D-galactosamine: polypeptide *N*-acetylgalactosaminyltransferase 13 (Accession no. XP_515839.1) and β-1,4-*N*-acetyl-galactosaminyl transferase 2 (Accession no. XP_548187.2) were identified with significant homology with PHB (Fig. 4A). Although it was very tempting to jump to the conclusion that PHB may have *O*-GlcNAc activity, however in both proteins PHB homologous region was not in the catalytic domain of these two enzymes indicating that it is unlikely that PHB has OGT activity. We speculate that these homologous regions in PHB and OGT family members are targeted by or interact with similar set of proteins and may have regulatory functions. In addition, homologous tyrosine motifs were also found between human OGT and central part of PHB containing Tyr114 and neighboring *O*-GlcNAc site that is Ser121; as well as between *Oryza* OGT and C-terminus part of PHB containing Tyr259 and neighboring *O*-GlcNAc site that is Thr258 (Fig. 4A). Subsequently we narrowed down our search to limit it to phospho tyrosine motifs in PHB and OGT family members. Interestingly pBLAST search for phospho tyrosine motifs in PHB limited to similar motifs in OGT family members revealed that tyrosine motifs in PHB containing Tyr114 and Tyr259 are very similar to tyrosine motifs present in various members of OGT family members (Fig. 4) suggesting that they may be targeted by similar tyrosine kinases and upon phosphorylation may interact with similar proteins. Recently OGT has been reported to interact with insulin receptor and insulin receptor induced tyrosine phosphorylation of OGT enhances OGT activity [15]. Therefore, we examined the tyrosine phosphorylation levels of OGT in cells co-transfected with OGT and PHB vectors in response to insulin. An increase in tyrosine phosphorylation of OGT was found with no change in OGT protein levels in cells transfected with PHB mutants (Fig. 3C). To ascertain whether increased *O*-GlcNAc modification of some proteins in the case of PHB mutants (Ty114Phe and Tyr259Phe) is due to increased OGT activity, *in vitro O*-GlcNAc assays were performed using immunoprecipitated OGT from insulin treated cells co-transfected with OGT and wild type PHB or tyrosine (Tyr114 and Tyr259) mutant-PHB. An increase in OGT enzyme activity was found consistent with increased tyrosine phosphorylation of OGT in comparison with controls (Fig. 3C). These data together suggests that increased *O*-GlcNAc modification of some proteins in cells transfected with PHB mutants were due to increased activity of OGT, most probably as a result of increased tyrosine phosphorylation of OGT.

### O-GlcNAc modification and tyrosine phosphorylation – A novel binary switch

To gain further insight into a potential global regulatory role of tyrosine phosphorylation on *O*-GlcNAc modification and *vice versa*, we analyze motifs around known sites of *O*-GlcNAc modified proteins, and known sites of phospho tyrosine containing proteins which are also known to be *O*-GlcNAc modified (Tables S1 and S2). Analysis of known *O*-GlcNAc modification sites in various proteins revealed that proline at −/+1 and/or −/+3 position from *O*-GlcNAc site(s) is the most favored residue for *O*-GlcNAc modification in a protein (Table S1). Therefore, to increase the possibility of finding clues for a potential interaction between *O*-GlcNAc modification and tyrosine phosphorylation within a protein, only those proteins were considered which met one of the following two sets of selection criteria: (i) having potential tyrosine phosphorylation site(s) in the close proximity of a known *O*-GlcNAc site(s) with proline residue(s) at −/+1 and/or −/+3 position of *O*-GlcNAc site(s) or (ii) having potential *O*-GlcNAc site(s) with proline residue(s) at −/+1 and/or −/+3 position in the close proximity of a known tyrosine phosphorylation site (in this category only those proteins were considered which are known to undergo *O*-GlcNAc modification however exact sites of modification are not known yet). A number of proteins were identified using each selection criteria (Tables S1 and S2) with strong association between these two modifications suggesting a potential interaction between these two dynamic modifications in many proteins (Table S3). Experiments are currently underway to further validate this hypothesis.

## Discussion

We provide evidence that PHB interacts with OGT in response to insulin and undergoes *O*-GlcNAc modification (Fig. 1). *O*-GlcNAc modification of PHB was further confirmed by metabolic labeling with GlcNAz sugar (tetraacetylated azide-modified *N*-acetylglucosamine) in C2C12 and NIH-3T3 cell lines (Fig. 1D). The acetyl groups improve cell-permeability of the azido-sugars and are removed by non-specific carboxyl esterases before sugars are incorporated into biosynthetic pathway.

Prediction of potential *O*-GlcNAc modification sites in PHB on the basis of known *O*-GlcNAc sites in several other proteins (Table S1) identified Ser101 (Proline at +3 position), Ser121 (Proline at −1 position) and Thr258 (Proline at +3 position) as potential *O*-GlcNAc modification sites (Fig. 4A). Interestingly, out of four tyrosine residues present in PHB, one of them (Tyr259) is adjacent to Thr258 within *O*-GlcNAc motif (^258^ **TY**LP^261^) and second (Tyr114) is flanked by two other potential *O*-GlcNAc modification sites i.e. Ser101 (^101^ **S**QLP^104^) and Ser121 (^120^P**S**ITT^124^) (Fig. 4A). *O*-GlcNAc modification of biotinylated peptide spanning potential *O*-GlcNAc modification sites by OGT (Fig. 2A) suggested that it could be *O*-GlcNAc sites *in vivo* too. Subsequently this is confirmed by site-directed mutagenesis and immunoblotting. Recently PHB homologue PHB2 was identified as *O*-GlcNAc modified protein in stress granules and it has been shown that *O*-GlcNAc modification is required for aggregation of untranslated mRNA into stress granules [22]. Out of two *O*-GlcNAc modification sites we have identified in PHB, Ser121 is conserved in PHB2 and corresponding residue in PHB2 is Ser135. Interestingly neighboring tyrosine residue (Tyr114) in PHB is also conserved in PHB2 and corresponding residue in PHB2 is Tyr128. Tyr128 in PHB2 has been identified as phosphorylation site during phospho-tyrosine profiling in cancer cells [23]. It would be interesting to know whether *O*-GlcNAc modification in PHB2 occurs at Ser135. It is possible that *O*-GlcNAc modification and tyrosine phosphorylation in PHB2 interact in the same way as in PHB.

In another study, PHB has been identified as a substrate for Akt however exact phosphorylation site remains to be determined [24]. Akt phosphorylates many proteins at serine/threonine residues that lie in Arg–Xaa–Arg–Xaa–Xaa–Ser/Thr motifs (where Xaa is any amino acid) and this motif is crucial for the specificity of Akt [25]. Analysis of PHB protein sequence reveals one potential motif ^254^RSRNIT ^258^ (R-x-R-x-x-S/T) for Akt. Interestingly Tyr259 in PHB is present at tandem position from Akt motif and Thr258 *O*-GlcNAc site is part of Akt consensus motif (R-x-R-x-x-S/T). This would imply that Thr258 may be subjected to *O*-GlcNAc modification or phosphorylation under different conditions and phosphorylation status of adjacent tyrosine residue (Tyr259) may play a regulatory role in this process.

**Figure 5:**
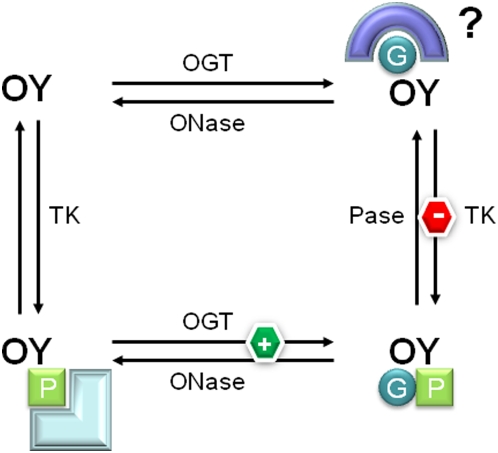
*O*-GlcNAc modification and tyrosine phosphorylation binary switch. Schematic diagram showing *O*-GlcNAc modifications at serine/threonine residues and tyrosine phosphorylation at neighboring tyrosine residues can potentially regulate each other in a binary fashion and subsequently may regulate interaction with different partners or attribute multiple functions to proteins (O - serine/threonine, Y – tyrosine, G - *O*-GlcNAc, P – phosphorylation, TK - tyrosine kinase, Pase - phosphatase, OGT - *O*-GlcNAc transferase, ONase - *O*-GlcNAcase).

Because PHB (as well as few other insulin signaling intermediates such as IRS, Akt, GLUT4 etc) undergoes both *O*-GlcNAc modification and tyrosine phosphorylation and these modification sites are either adjacent or in the close proximity to each other we hypothesize that *O*-GlcNAc modification and tyrosine phosphorylation may be a previously unidentified novel binary switch. This would imply that these modifications may interact and regulate each other under different conditions, provide read-out for different interacting partners, attribute multiple functions to the same protein and may play an important role in cell signaling cascades (Fig. 5). Using biotinylated peptides spanning potential *O*-GlcNAc modification and tyrosine phosphorylation sites we showed that they indeed affect each other (Fig. 2B). This is further confirmed *in vivo* by substituting tyrosine phosphorylation and *O*-GlcNAc modification sites by site-directed mutagenesis (Fig. 2C–D). In addition, an up regulation of *O*-GlcNAc modification of some proteins was observed in the absence of tyrosine phosphorylation sites in PHB in response to insulin (Fig. 2). Because PHB and OGT both interacts with insulin receptor, undergoes tyrosine phosphorylation and contain similar tyrosine motifs it is possible that they may be part of a protein complex which may also include kinases and phosphatases, and under such condition loss of a competitive tyrosine phosphorylation sites in PHB may affect tyrosine phosphorylation and activity of OGT. Indeed this is exactly what we found in our case and is consistent with recent report by Whelan *et al* (2008). However, both OGT and PHB contain similar phospho tyrosine motifs therefore recruitment and subsequent modulation by other phospho tyrosine binding (PTB) domain containing proteins cannot be ruled out and warrant further investigation.

Using two stringent selection criteria (as discussed above) a number of proteins were identified with potential tyrosine phosphorylation sites in the proximity of known *O*-GlcNAc sites or *vice versa* (Table S1 and S2). In both cases, a strong association was found between presence of proline residues with *O*-GlcNAc site (59–93%), as well as *O*-GlcNAc and tyrosine phosphorylation sites (71–87%) (Table S3). Given the fact that our knowledge regarding *O*-GlcNAc sites in various proteins are very limited, this high degree of association between *O*-GlcNAc modification and tyrosine phosphorylation in various proteins suggests a functional link between these two dynamic modifications in the regulation of various cellular events. We speculate that presence of multiple proline residues around *O*-GlcNAc/phospho tyrosine cassette play an important role not only in *O*-GlcNAc modification but also in the recruitment of various interacting partners especially proteins/protein domains which are known to bind phosphorylated residues adjacent to proline residues. In addition, phosphorylation status of tyrosine may play a regulatory role in the cross-talk between serine/threonine phosphorylation and *O*-GlcNAc modification. This study offers a novel mechanistic insight in cell signaling pathways.

## Materials and Methods

### Cell culture, site directed mutagenesis and transfection

For all cell based experiments cells were grown in 100 mm culture dish in RPMI-1640 (ATCC, USA) complete growth media containing 10% FBS, 1% antibiotic-antimycotic at 37°C. OGT construct was obtained from Prof. Gerald Hart, University of John Hopkins. The pCMV6-XL5 vector containing human PHB clone (Reference sequence XM_056688) was obtained from Origene technologies, USA. PHB mutants were made using site-directed mutagenesis kit (Stratagene, USA) following manufacturer's instructions. The following primers were used for generating PHB mutants: Y114F, Forward - 5′ CAGCATCGGAGAGGACTTTGATGAGCGTGTGC 3′ and reverse - 5′ GCACACGCTCATCAAAGTCCTCTCCGATGCTG 3′; Y259F: Forward -5′ TCTCGGAACATCACCTTCCTGCCAGCGG 3′ and reverse - 5′ CCGCTGGCAGGAAGGTGATGTTCCGAGA 3′; S121A Forward - 5′ AGCGTGTGCTGCCGGCCATCACAACTGAG 3′ and reverse - 5′ CTCAGTTGTGATGGCCGGCAGCACACGCT 3′; T258I Forward - 5′ CTCTCGGAACATCATCTACCTGCCAGCGG 3′ and reverse - 5′ CCGCTGGCAGGTAGATGATGTTCCGAGAG 3′. Authenticity of all constructs was confirmed by DNA sequencing at Cancer Care Manitoba. Transfections were performed using lipofectamine (Invitrogen, USA), according to manufacturer's protocol. After 48 hrs of transfection cells were serum starved and treated with insulin (100 nM) for 20 minutes. Cell lysates were prepared in lysis buffer (50 mM Tris-Cl, pH 7.4) containing protease and phosphatase inhibitors [26] and subsequently processed for immunoprecipitation using specific antibodies as described below.

### Recombinant protein and peptides

Recombinant human His-tagged PHB (His-PHB) expressed in *Escherichia coli* and purified by His-affinity column was obtained from AmProx Inc. (Carlsbad, CA, USA). Custom made PHB^105–123^ and PHB2^250–268^ peptides (Pep I and Pep II) were chemically synthesized as 19mer peptide amidated at C-terminus and biotinylated at N-terminus. The correct peptides were obtained in greater than 90% yield and were homogeneous after purification as confirmed by mass and analytical HPLC.

### Immunoprecipitation and Western blot

Immunoprecipitation of PHB and OGT was performed using Dynabeads Protein A and DynaMag (Invitrogen, USA) according to manufacturer's protocol. In brief, to 1 ml of cell lysate (adjusted for equal amount of protein), 10 µl of rabbit polyclonal anti-PHB antibody or monoclonal OGT antibody were added and incubated overnight on a rotating device at 4°C. At the end of the incubation, 25 µl of Dynabeads protein A suspension were added to each tube and further incubated for 2 h. Subsequently, pellets were washed (5×) in ice cold PBS, resuspended and used for in vitro assays and Western blot. Immunoprecipitated proteins were separated on 12% SDS-PAGE and transferred to nitrocellulose membrane for Western blotting as described earlier [26].

### Confocal microscopy

RINm5F cells were cultured in chambered slides, fixed with 4% formaldehyde in PBS and incubated with antibodies to OGT (Santa Cruz Biotechnology, USA), and PHB (Cell Signaling, USA) at a concentration of 1 µg/ml in PBS containing 0.1% BSA. Immune complexes were detected with TRITC and FITC conjugated antibodies to mice and rabbit (Sigma, Oakville, ON) respectively.

### O-GlcNAc metabolic labeling of PHB

*O*-GlcNAc metabolic labeling was performed using Click-iT GlcNAz metabolic glycoprotein labeling reagent (tetraacetylated *N*-azidoacetylgalactosamine and glucosamine) from Invitrogen, USA. In brief, cultures were seeded at 2.5×10^5^ cells/ml in 100 mm tissue culture dish and allowed to grow for 72 hrs in the presence of 40 µM tetraacetylated azido-sugar. Cell lysates were prepared with or without insulin treatment in lysis buffer (1% SDS, 50 mM Tris-HCl, pH 8.0) containing protease and phosphatase inhibitors [26]. PHB was immunoprecipitated using rabbit anti-PHB antibody (Cell Signaling, USA) and Dynabeads Protein A (Invitrogen, USA) and subsequently analyzed with Click-iT Protein Analysis Detection Kit (Invitrogen, USA) according to manufacturer's protocol.

### In vitro phosphorylation assay

PHB synthetic peptides (2 µg) (Pep I - Biotin-^105^RIFTSIGED**Y**DERVLP**S**IT^123^-NH2 and Pep II - Biotin-^250^QLSRSRNI**TY**LPAGQSVLL^268^-NH2, amidated at C-terminus and biotinylated at N-terminus) were incubated with 20 ng of recombinant active insulin receptor in kinase buffer (50 mM Tris-HCl, pH 7.5, 10 mM MgCl2, 50 µM ATP) for 20 min at 30°C. At the end of assays, 10 µl of loading buffer was added to each tube and analyzed on 16% SDS-PAGE, transferred to nitrocellulose membranes and processed for Western blot as described above. To study the effect of *O*-GlcNAc modification on tyrosine phosphorylation peptides were first immobilized on streptavidin-agarose and then processed for *in vitro O*-glycosylation assay using immunoprecipitated OGT and UDP-GlcNAc. At the end of incubation, tubes were transferred to the ice and subsequently washed (3×) to remove reaction mixture and finally resuspended in 50 mM Tris-Cl buffer pH 7.4 for subsequent use in an *in vitro* kinase assay using catalytic domain of insulin receptor and ATP. Subsequently peptides were analyzed by gel-electrophoresis and Western blotting as described previously [27].

### In vitro O-GlcNAc assays

PHB peptides (Pep I and Pep II) containing Ser121 and Thr258 were incubated with immunoprecipitated OGT in assay buffer (0.50 mM Na-Cacylodate pH 6.5, UDP-GlcNAc, 0.250 mM NaF) in a total reaction volume of 40 µl for 2 h at 30°C [15]. Reaction was stopped by adding 10 µl of SDS-PAGE loading buffer and subsequently analyzed with gel-electrophoresis and immunoblotting using monoclonal anti–*O*-GlcNAc antibody. In some cases, biotinylated peptides were immobilized on streptavidin-agarose and subsequently used for *in vitro* assay as described above.

## Supporting Information

Table S1 Protein sequences having potential tyrosine phosphorylation site in the vicinity of known *O*-GlcNAc site.
(0.09 MB PDF)

Table S2 Protein sequences having potential *O*-GlcNAc site in the vicinity of known tyrosine phosphorylation known.
(0.12 MB PDF)

Table S3 Summary of potential *O*-GlcNAc and phospho tyrosine binary switch.
(0.14 MB PDF)
