# Supplementary material for: Interaction between O-GlcNAc Modification and Tyrosine Phosphorylation of Prohibitin: Implication for a Novel Binary Switch
Source: PLoS One. 2009 Feb 24;4(2):e4586. doi: 10.1371/journal.pone.0004586 (PMC2642629; doi:10.1371/journal.pone.0004586)
Supplement: Table S1 — Protein sequences having potential tyrosine phosphorylation site in the vicinity of known O-GlcNAc site. (0.09 MB PDF) [file pone.0004586.s001.pdf]

Table S1. List of small sequences of short listed proteins using selection criteria # 1 (i.e. proteins with known *O*-GlcNAc modification site(s) (**Sg/Tg**) having proline at either -/+1 and/or -/+3 position (**P**), with potential tyrosine phosphorylation site (**Y**) in the vicinity) showing known *O*-GlcNAc site(s) with potential tyrosine phosphorylation site(s) in the vicinity. Similar residues in prohibitin are also shown.

Protein	Sequence
Bassoon	1414 SgPSgTgSgSgTIHSgYGQPPTT 1430
Bassoon	1932 SVTgDTALPGQSSGPFYSPR 1950
Bassoon	2120 HGSgGSgGGPDLVQYQPQHGPGLSgAPQ 2144
Bassoon	3213 SgSgVSgQSgPAPTgYPSgDSgHYTSgL 3232
CREB coactivator CRTC2 (TORC2)	65 SHYGGSgLPNVNQIGCGLAEFQSPLHSP 91
CREB Coactivator CRTC2 (TORC2)	170 SSgDSgALHTSVMNPNPQDTYPGPTPPSVLPS 199
Estrogen receptor beta	55 NYSVPSSgTGNLEGGPV 70
Microtubule-associated Protein 1B	2018 TTTKTTRSpPDTSAYCYE 2034
Neurofilament L	38 SAYSSYSAPVSgSgSgLSgVR 54
Neurofilament L	31 SGYSgTAR 37
Neurofilament M	43 GSPSgTgVSgSgSgYKR 55
Piccolo	2941 TTGPYPETgR 2949
Piccolo	2949 QVISGISTPQYSgTgAR 2966
Ponsin	1189 TPVDYIDLPYSgSgSgPSR 1204
RNA-binding motif protein 14	275 AQPSgVSgLGAPYR 296
Vimentin	51 SLYASgSPGGV 60
Prohibitin	101 SQLPRIFTSIGEDYERVLPS………NITYLP 261
	114 259

Note: Additional potential *O*-GlcNAc sites with proline at -1 or +3 positions are shown as bold letters (**S/T**).
