# Supplementary material for: Interaction between O-GlcNAc Modification and Tyrosine Phosphorylation of Prohibitin: Implication for a Novel Binary Switch
Source: PLoS One. 2009 Feb 24;4(2):e4586. doi: 10.1371/journal.pone.0004586 (PMC2642629; doi:10.1371/journal.pone.0004586)
Supplement: Table S2 — Protein sequences having potential O-GlcNAc site in the vicinity of known tyrosine phosphorylation known. (0.12 MB PDF) [file pone.0004586.s002.pdf]

Table S2. List of small sequences of short listed proteins using selection criteria # 2 (i.e. protein with known tyrosine phosphorylation site (**pY**) with proline at either -/+1 and/or -/+3 position (P) of a potential *O*-GlcNAc site (**S/T**) in the vicinity) showing known tyrosine phosphorylation site with potential *O*-GlcNAc site(s) in the vicinity.

Protein	pY site	Sequence
AnkyrinG	533	526 NAATTSGpYTPLHLSA 540
β-amyloid precursor protein	757	750 SKMQQNGpYENPTYKF 764
β-amyloid precursor protein	762	755 NGYENPTpYKFFEQMQ 769
Insulin receptor substrate 1	662	655 QRVDPNGpYMMMSPSG 669
Insulin receptor substrate 1	989	982 VPSSRGDpYMTMQMSC 996
Keratin 8	25	18 RAFSSRSpYTSGPGSR 32
Microtubule-associated Protein 1B	1062	1055 AGGAEEQpYGFLTTPT 1069
Microtubule-associated Protein 1B	1336	1329 GSAGHTPpYYQSPTDE 1343
Microtubule-associated Protein 1B	1796	1789 PRESSPLpYSPTFSDS 1803
Microtubule-associated Protein 1B	1830	1823 DAASAEPpYGFRASVL 1837
Microtubule-associated Protein 1B	1921	1914 KSPSDSGpYSYETIGK 1928
Phosphatidylinositol kinase 3	425	418 KTINPSKpYQTIRKAG 432
Rho GDP-dissociation inhibitor	144	137 VKIDKTDpYMVGSYGP 151
Tubilin α	224	217 LDIERPTpYTNLNRLI 231
Tubulina α	357	350 GFKVGINpYQPPTVVP 364
Annexin1	38	31 PGSAVSPpYPTFNPSS 45
Annnexin1	229	222 TILTTRSpYPQLRRVF 236
Dyenin	3377	3370 NYMSNPSpYNYEIVNR 3384
ATP-binding cassette, sub-family C	1508	1501 DKGEIQEpYGAPSDLL 1515
Heat-shock protein 70	41	34 GNRTTPSpYVAFTDTE 48
Insulin receptor substrate 2	814	807 PRSYKAPpYTCGGDSD 821
Keratin 18	23	16 GSVQAPSpYGARPVSS 30
Nuclear factor-kappa B 105	59	52 QRGFRFRpYVCEGPSH 66
Piccolo	1824	1817 LPTAVSLpYSPTDEQS 1831
Phosphatidylinositol kinase 3	440	433 KTGERCLpYMWPSVPD 447
Ponsin	134	127 SSLAAKGpYRSVHPNL 141
RNA polymerase II	1860	1853 YTPTSPKpYSPTSPKY 1867
RNA polymerase II	1909	1902 YTPTSPKpYSPTSPTY 1916
RNA polymerase II	1923	1916 YSPTSPKpYSPTSPTY 1930
Vimentin	53	46 PSTSRSLpYASSPGGV 60
Prohibitin	114	107 FTSIGEDpYDERVLPS 121
Prohibitin	259	252 SRSRNITpYLPAGQSV 266

Note: Above listed proteins are known to be *O*-GlcNAc modified however in majority of the cases *O*-GlcNAc modification sites are not known yet.
