# Supplementary material for: Interaction between O-GlcNAc Modification and Tyrosine Phosphorylation of Prohibitin: Implication for a Novel Binary Switch
Source: PLoS One. 2009 Feb 24;4(2):e4586. doi: 10.1371/journal.pone.0004586 (PMC2642629; doi:10.1371/journal.pone.0004586)
Supplement: Table S3 — Summary of potential O-GlcNAc and phospho tyrosine binary switch. (0.14 MB PDF) [file pone.0004586.s003.pdf]

Table S3. Salient features of protein sequences listed in Table S1 and Table S2 containing potential O-GlcNAc and phospho tyrosine binary switch.

Selection criteria #1<br>(No. of sequence listed - 17)	Selection criteria #2<br>(No. of sequence listed - 30)
1. Number of proteins with a proline residue at -/+1 and/or -/+3 position from known *O*-GlcNAc site: 10 (59%).<br>respectively.<br>2. Proline residue within -/+ 5 residue from known *O*-GlcNAc site: 14 (82.%)<br>3. Number of proteins containing potential tyrosine phosphorylation site within -/+ 5 residue of known OGN site: 12 (71%).	1. Number of proteins with a proline residue at -/+1 and/or -/+3 position from potential *O*-GlcNAc site: 28 (93%).<br>2. Proline residue within -/+ 5 residue from a known phospho tyrosine site: 26 (87%).<br>3. Number of potential *O*-GlcNAc sites within -/+ 5 residues of known tyrosine phosphorylation site: 26 (87%).
